# Characterizing longitudinal white matter development during early childhood

**DOI:** 10.1007/s00429-014-0763-3

**Published:** 2014-04-08

**Authors:** Douglas C. Dean, Jonathan O’Muircheartaigh, Holly Dirks, Nicole Waskiewicz, Lindsay Walker, Ellen Doernberg, Irene Piryatinsky, Sean C. L. Deoni

**Affiliations:** 1 Advanced Baby Imaging Laboratory, School of Engineering, Brown University, Providence, RI 02912 USA; 2 Department of Neuroimaging, King’s College London, Institute of Psychiatry, De Crespigny Park, London, SE5 8AF UK

**Keywords:** Brain development, White matter development, Magnetic resonance imaging, Myelin water fraction

## Abstract

Post-mortem studies have shown the maturation of the brain’s myelinated white matter, crucial for efficient and coordinated brain communication, follows a nonlinear spatio-temporal pattern that corresponds with the onset and refinement of cognitive functions and behaviors. Unfortunately, investigation of myelination in vivo is challenging and, thus, little is known about the normative pattern of myelination, or its association with functional development. Using a novel quantitative magnetic resonance imaging technique sensitive to myelin we examined longitudinal white matter development in 108 typically developing children ranging in age from 2.5 months to 5.5 years. Using nonlinear mixed effects modeling, we provide the first in vivo longitudinal description of myelin water fraction development. Moreover, we show distinct male and female developmental patterns, and demonstrate significant relationships between myelin content and measures of cognitive function. These findings advance a new understanding of healthy brain development and provide a foundation from which to assess atypical development.

**Electronic supplementary material:**

The online version of this article (doi:10.1007/s00429-014-0763-3) contains supplementary material, which is available to authorized users.

## Introduction

Human brain development is a multifaceted process that begins in utero and continues throughout childhood and into early adulthood. Despite this protracted timeline, postnatal neurodevelopment during the first 5 years of life is one of the most active stages of brain development (Pujol et al. 2006). During this period neural migration, synapse generation, dendritic sprouting, axonal pruning, and the elaboration of the myelin sheath (myelination) shape, organize, and provide the neural architecture necessary for behavioral and cognitive functioning (Durston and Casey 2006). While each of these processes is essential for healthy brain function, the establishment of the myelinated white matter is integral to establishing the efficient brain communication pathways that are essential for higher order function (Fields 2008). Although myelination is believed to mirror behavioral development (Pujol et al. 2006), with the myelination of sub-serving brain networks occurring alongside the onset and refinement of behavioral and cognitive functions, the relationships between myelination and functional development remain poorly understood. Furthermore, many behavioral and psychiatric disorders are now believed to emerge during early neurodevelopment (Hüppi 2008) and have been associated with aberrant white matter development and myelination (Fields 2008). Thus, improved understanding of typical myelin development during infancy and early childhood is central to provide valuable insight into the processes that underline both typical and atypical development.

Advanced magnetic resonance imaging (MRI) techniques have greatly contributed to our understanding of the gross and microstructural changes associated with brain maturation (Giedd and Rapoport 2010). For example, the temporal changes in the gray/white matter contrast in conventional *T*_1_- and *T*_2_ -weighted MR images has been used to illustrate the dynamic patterns of neurodevelopment (Paus et al. 2001). Diffusion tensor (DT)-MRI parameters, including mean and radial diffusivity (MD and RD, respectively), and fractional anisotropy (FA), which describe local measures of water diffusion and anisotropy, have been shown to change throughout childhood and adolescence (Lebel et al. 2008). Magnetization transfer (MT) imaging, believed to be sensitive to myelin content, has additionally been used to examine the in vivo changes of white matter myelination (Rademacher et al. 1999). While alterations of these MR parameters are often attributed to the emergence and maturation of myelin, they do not directly measure myelin (Paus et al. 2001) and are influenced by a broad range of other microstructural changes (Jones et al. 2013).

Beyond the technological difficulty in non-invasively imaging myelination, few imaging studies have examined longitudinal neurodevelopment across the first years of life. Rather, prior studies have either been cross-sectional in nature (Giedd et al. 1996; Casey et al. 2005) and/or have focused on only the first 1–2 years of life (Gilmore et al. 2012; Sadeghi et al. 2013), or after the age of 4 (Giedd et al. 1999; Lenroot et al. 2007; Lebel and Beaulieu 2011). Although cross-sectional studies are informative, they are inherently insensitive to individual differences, an important aspect in understanding the heterogeneity associated with both normative development, as well as in developmental disorders (Casey et al. 2005). Longitudinal study designs, on the other hand, afford a more thorough characterization of brain development. Through the acquisition of multiple measurements from the same individual, longitudinal analyses allow individual differences to be examined (Casey et al. 2005) and are, therefore, critical to understanding the developmental trajectory of brain development. However, as current longitudinal MRI studies have been limited to infancy (Gilmore et al. 2012; Sadeghi et al. 2013), or older childhood and adolescence (Giedd et al. 1999; Lenroot et al. 2007; Lebel and Beaulieu 2011), these studies fail to encompass the most rapid and dynamic period of neurodevelopment (Pujol et al. 2006), which also coincides with the emergence of many developmental neuropsychiatric disorders, including autism spectrum and attention deficit/hyperactivity disorder.

Here, we aimed to perform the first longitudinal examination of normative brain development in healthy, typically developing children between 2.5 months and 5.5 years. More specifically, we examined the rapid, nonlinear progression of myelination using a quantitative multicomponent relaxometry (MCR) technique that is sensitive to the water trapped between the lipid bilayers of the myelin sheath (MacKay et al. 1994; Deoni et al. 2008). Repeated MRI scans were acquired using the rapid mcDESPOT (multicomponent driven equilibrium single pulse observation of *T*_1_ and *T*_2_) (Deoni et al. 2008) MCR technique in a large cohort of 108 typically developing children. Longitudinal developmental trajectories from 28 brain regions and major white matter pathways are presented. We utilize a nonlinear mixed effects modeling framework and a four-parameter Gompertz function (Dean et al. 2014a) to explicitly model the nonlinear growth patterns of the myelin water fraction (VF_M_), a surrogate measure of myelin content, for each individual (random effects) and the overall population (fixed effects). Parameter estimates from this nonlinear fitting lead to the characterization of a normative model of VF_M_ development, which can then be used to reconstruct typical VF_M_ growth and growth rate patterns. Finally, modeled trajectories, coupled with longitudinal scores of cognition and behavior, were examined to identify relationships between VF_M_ and functional development. Quantitative analysis of these data provides further insight into the processes of brain maturation and its relationship to cognitive evolution, and provides a foundation for future studies of atypical development.

## Methods

### Subjects

Subjects for this study consisted of 108 healthy, typically developing children recruited to take part in an ongoing longitudinal study investigating the relationships between myelin maturation and behavioral development (Deoni et al. 2012). Parental consent was obtained in accordance to ethics approval from the host institution’s Institutional Review Board. Inclusion criteria consisted of uncomplicated (i.e., no preeclampsia, and APGAR scores >8) singleton birth between 37 and 42 weeks; no abnormalities on fetal ultrasound; no exposure to alcohol or illicit drugs during pregnancy; no familial history of major psychiatric or depressive illnesses; no diagnosis of major psychiatric, depressive or learning disorder in the participant; and no pre-existing neurological conditions or major head trauma.

Subjects were composed of 51 females and 57 males that were between 70 and 1,928 days (GC, gestationally corrected to a 40 week gestation) of age at recruitment. An age-dependent scanning schedule was used to determine when children would return for follow-up MRI sessions. Children under 2 years of age returned every 6 months, while children over 2 years of age returned yearly (Dean et al. 2014b). Additional demographic information is provided in Supplementary Table 1. No statistical differences between birth weight, birth height, and gestational period were found between the age groups.

### Image acquisition

Scanning of children under 4 years of age was performed during natural (i.e., non-sedated) sleep (Dean et al. 2014b). Children over this age were scanned while watching a favorite movie or TV show. Imaging slew rates and gradient amplitudes were reduced to approximately 30 and 75 % of their maximal values to reduce the acoustic noise levels of the MRI scanner and ensure sleeping children remained asleep for the duration of the scan. Additional passive measures, including electrodynamic headphones (MR Confon, Germany) and a removable sound-insulating foam insert (Ultra Barrier HD Composite, UltraBarrier USA) that conformed to the inside of the scanner bore, provided further acoustic noise attenuation. To reduce subtle body movement, children were swaddled with appropriately sized MedVac vacuum immobilization bags (CFI Medical Solutions, USA).

All image data were acquired on the same 3T Siemens Tim Trio MRI scanner with a 12-channel head RF coil array. Optimized pediatric mcDESPOT protocols previously developed for varying head size and age range were used (Deoni et al. 2012). In general, the mcDEPSOT imaging protocol consists of 8 *T*_1_-weighted spoiled gradient echo (SPGR or spoiled FLASH) images, 2 inversion-prepared (IR)-SPGR images, and 16 *T*_1_/*T*_2_-weighted balanced steady–steady state free precession (bSSFP or TrueFISP) images. SPGR and bSSFP images were acquired at varying flip angles, while the bSSFP images were collected at two-phase increments (0° and 180°). Field of view and the image matrix size were adjusted so that whole-brain coverage was achieved while maintaining a consistent 1.8 × 1.8 × 1.8 mm^3^ voxel resolution. Total acquisition time for all protocols was <30 min. Additional information about the imaging parameters used is included as supplementary information (Supplementary Table 2).

In addition to the mcDESPOT data, a higher resolution (1.5 × 1.5 × 1.5 mm^3^) *T*_1_-weighted image and resting state functional MRI data were acquired if the child remained asleep and cooperative.

### VF_M_ measurements

Following successful MR data acquisition, the 26 individual SPGR, IR-SPGR, and bSSFP images for each participant were linearly co-registered to account for subtle head movement and non-parenchyma voxels removed using a deformable model approach (Smith et al. 2004). VF_M_ values were then calculated at each image voxel by fitting the SPGR and bSSFP data to a 3-pool tissue model that estimates the volume fractions and relaxation times for intra/extra-axonal water, myelin-associated water, and non-exchanging free water (Deoni et al. 2013). Quantification of the properties from these microstructural water compartments distinguishes each component and therefore provides unique information about the underlying microstructure. Additional corrections for radio-frequency flip angle (*B*_1_) and main magnetic field (*B*_0_) inhomogeneities were also performed (Deoni 2010).

### Registration of longitudinal measurements

Independently registering longitudinal measurements to a common template may introduce subtle inconsistencies when not accounting for the repeated regularity of subject-specific measurements (Aubert-Broche et al. 2013). To minimize this possible source of variability, a longitudinal registration pipeline was developed to align each individual’s measurements into a common analysis space. This registration was performed using the high flip angle SPGR image with the resulting transformations subsequently applied to the quantitative VF_M_ maps.

For each subject, a *T*_1_-weighted template was created from the acquired longitudinal time points using symmetric diffeomorphic normalization and a cross-correlation similarity metric (Avants et al. 2008). An initial rigid registration between the subject’s high flip angle SPGR images and a study-specific template previously created from *T*_1_-weighted data of children of the same age range and approximately in the space of the MNI template (Deoni et al. 2012) was performed to bring the images into a rough alignment. Next, a subject-specific template was created from these roughly aligned SPGR images using the ANTs buildtemplateparallel.sh script that is included in the ANTs package. The subject-specific template was then nonlinearly registered to the study template using symmetric diffeomorphic normalization. Finally, individual parameter maps for each time point were transformed into the overall study template by concatenating the transformations from native space to the subject-specific template space and from subject-specific template space to the overall study template in a single interpolation step (Supplementary Fig. 1).

Following successful registration of each individual dataset, VF_M_ images were smoothed with a conservative 3-mm (full width at half maximum) Gaussian kernel to accommodate subtle structural variations among the individual subjects that were not handled by the registration procedure.

### Longitudinal developmental trajectories

While there exists high individual developmental variation, distinct patterns of structural development can be observed and provide insight to brain maturation. For this reason we constructed longitudinal developmental trajectories of VF_M_ from our cohort of subjects. Anatomically co-registered masks, obtained from the MNI database (Mazziotta et al. 2001) and the Johns Hopkins University DTI-based white matter atlas (Mori et al. 2009), were applied to the individual VF_M_ data. In total, 28 regions were examined (detailed in Supplementary Fig. 2). For each longitudinal time point and anatomical region, mean VF_M_ values were extracted and plotted against the subject’s gestation-corrected age.

### Modeling longitudinal VF_M_ development

Nonlinear mixed effects modeling was used to characterize VF_M_ development due to its ability to account for repeated measurements from the same individual, and non-equally distributed data (Lindstrom and Bates 1990). Modified Gompertz growth curves of the form:

1 $${\text{VF}}_{\text{M}} = \alpha \,\exp \,( - \exp (\beta - \gamma_{\text{t}} ) + \delta_{\text{t}} ),$$

were fit to the developmental trajectories from the 28 regions (MATLAB 2013a, Natick, MA). This sigmoidal model was chosen to describe the nonlinear growth patterns of early neurodevelopment as this model has previously been shown to best represent VF_M_ development in a cross-sectional cohort and offers informative parameterization of the developmental trajectory (Dean et al. 2014a, b); where *α* controls for the overall size of the curve, *β* controls for the initial lag of the trajectory, and the parameters *γ* and *δ* both correspond to growth rates. Supplementary Fig. 3 illustrates the effect that each parameter has on an example model growth curve.

Within this model formulation, the set of parameters, (*α*, *β*, *γ*, *δ*), corresponds to the sum of the fixed and random effects. The set of parameters corresponding to the fixed effects characterize the overall population trajectory, while parameters that describe the random effects are estimated for each individual and allow the estimation of subject-specific growth curves (Lindstrom and Bates 1990).

In addition to modeling population (fixed effects) and individual (random effects) growth, the growth rate of VF_M_ during this time period can also be examined. Using the Gompertz growth parameter estimates from above, we compute the rate of change of VF_M_ by taking the time derivative of Eq. (1),

2 $$\partial_{\text{t}} {\text{VF}}_{\text{M}} = \alpha \,\exp \,( - \exp (\beta - \gamma_{\text{t}} ))[\gamma \,\exp (\beta - \gamma_{\text{t}} ) + \delta ]$$

From this equation and using the estimates of the fixed and random effect growth parameters, both population and individual growth rates curves can be calculated.

### Sex-related VF_M_ developmental differences

As gender-related developmental differences are often reported to occur during early neurodevelopment (Lenroot et al. 2007), we sought to examine if gender differences in VF_M_ were apparent. Nonlinear mixed model fits of the regional growth trajectories to the modified Gompertz equation (Eq. 1) were independently performed on gender-separated longitudinal data. *F*-statistics were used to compare the residuals of the nonlinear fitting of sex-separated and sex-combined developmental trajectories, while two-sided *t*-statistics were used to compare male and female modified Gompertz parameter estimates. Significance was defined to be *p* < 0.05 corrected for the familywise error rate using Holm–Bonferroni method (Holm 1979).

### Investigation of VF_M_ development and cognition

To elucidate the association between myelination and cognitive development, we performed linear regressions between longitudinal VF_M_ data and repeated measures of age-appropriate assessments of motor control, language, and visual reception. Within 1 week of successful acquisition of MRI data, children were administered the Mullen Scales of Early Learning (Mullen 1995) by a trained researcher. This developmental assessment battery provided five scales of cognitive and behavioral ability (gross motor, fine motor, expressive language, receptive language, and visual reception) from which we could interrogate the inherent relationship between structural and functional development. Due to the appropriate age range for the exam and a known ceiling effect inherent to the Mullen battery (Mullen 1995; O’Muircheartaigh et al. 2013), we restricted our analysis to a subset of 69 datasets in which the final MRI scan was acquired from children under 1080 GC days (or *N* months). (Supplementary Table S3).

For each individual’s raw Mullen’s scores, an index of cognitive change was computed using the following formula,

3 $$\varDelta_{\text{M}} = \frac{{M_{\text{f}} - M_{\text{i}} }}{{M_{\text{f}} + M_{\text{i}} }},$$

where *Μ*_f_ and *M*_i_ represent the raw Mullen’s scores measured at the subject’s final and initial cognitive assessment, respectively, and Δ_Μ_ represents the normalized measure of cognitive change between these two time points. Similarly, for each region of interest, subject-specific indices of VF_M_ change were computed,

4 $$\varDelta_{{{\text{VF}}_{\text{M}} }} = \frac{{{\text{VF}}_{\text{M}}^{\text{f}} - {\text{VF}}_{\text{M}}^{\text{i}} }}{{{\text{VF}}_{\text{M}}^{\text{f}} + {\text{VF}}_{{{\text{M}}_{\text{i}} }}^{\text{i}} }},$$

where ${\text{VF}}_{\text{M}}^{\text{f}}$ and ${\text{VF}}_{\text{M}}^{\text{i}}$ represent final and initial VF_M_ values calculated from the subject-specific VF_M_ Gompertz growth curves (Eq. 1) at the subject’s final and initial age, respectively, and $\varDelta_{{{\text{VF}}_{\text{M}} }}$ represents the normalized change in modeled VF_M_ between the final and initial time points. Relationships between $\varDelta_{{{\text{VF}}_{\text{M}} }}$ and each Δ_M_ (gross and fine motor, expressive and receptive language, and visual reception) as well as their interactions with mean age, were tested using a multivariate general linear model (GLM), correcting for the difference between final and initial ages, and the mean age of the time points. The inclusion of these two covariates within this GLM was important as both raw cognitive measures and VF_M_ are mediated by age (O’Muircheartaigh et al. 2013). In addition, combining all the cognitive scales into a single GLM allowed us to partially account for the correlation between each of the cognitive measures (Mullen 1995). This model was tested non-parametrically using permutation testing (FSL’s randomize) and significance was defined to be *p* < 0.05, corrected for the familywise error rate.

## Results

**Fig. 1:**
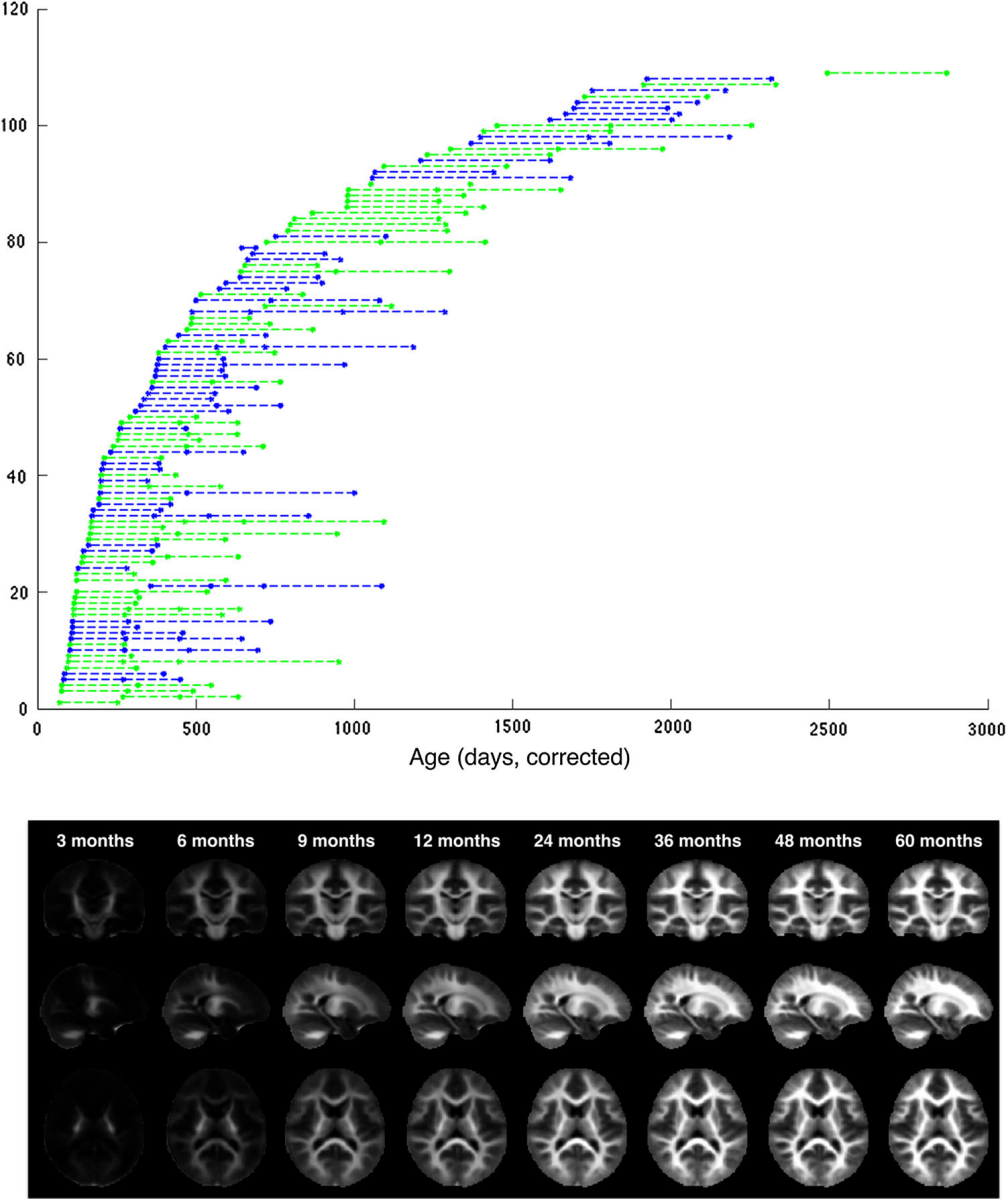
*Top row*: ages of the 108 subjects at each scan.* Each row* denotes an individual subject and the repeated measurements are connected with a *dashed line*. Males (*blue*) and females (*green*) are additionally distinguished. *Bottom row*: mean mcDESPOT VF_M_ maps at 3, 6, 9, 12, 24, 36, 48, and 60 months of age illustrating the development of myelinated white matter throughout the brain

A total of 260 mcDESPOT MRI datasets were acquired from the 108 participants: 73 subjects were scanned twice, 26 were scanned three times, and 9 were scanned 4 times. Matched coronal, sagittal, and coronal slices through the mean VF_M_ maps for representative age groups are shown in Fig. 1 and illustrate the development of myelinated white matter throughout the brain.

**Fig. 2:**
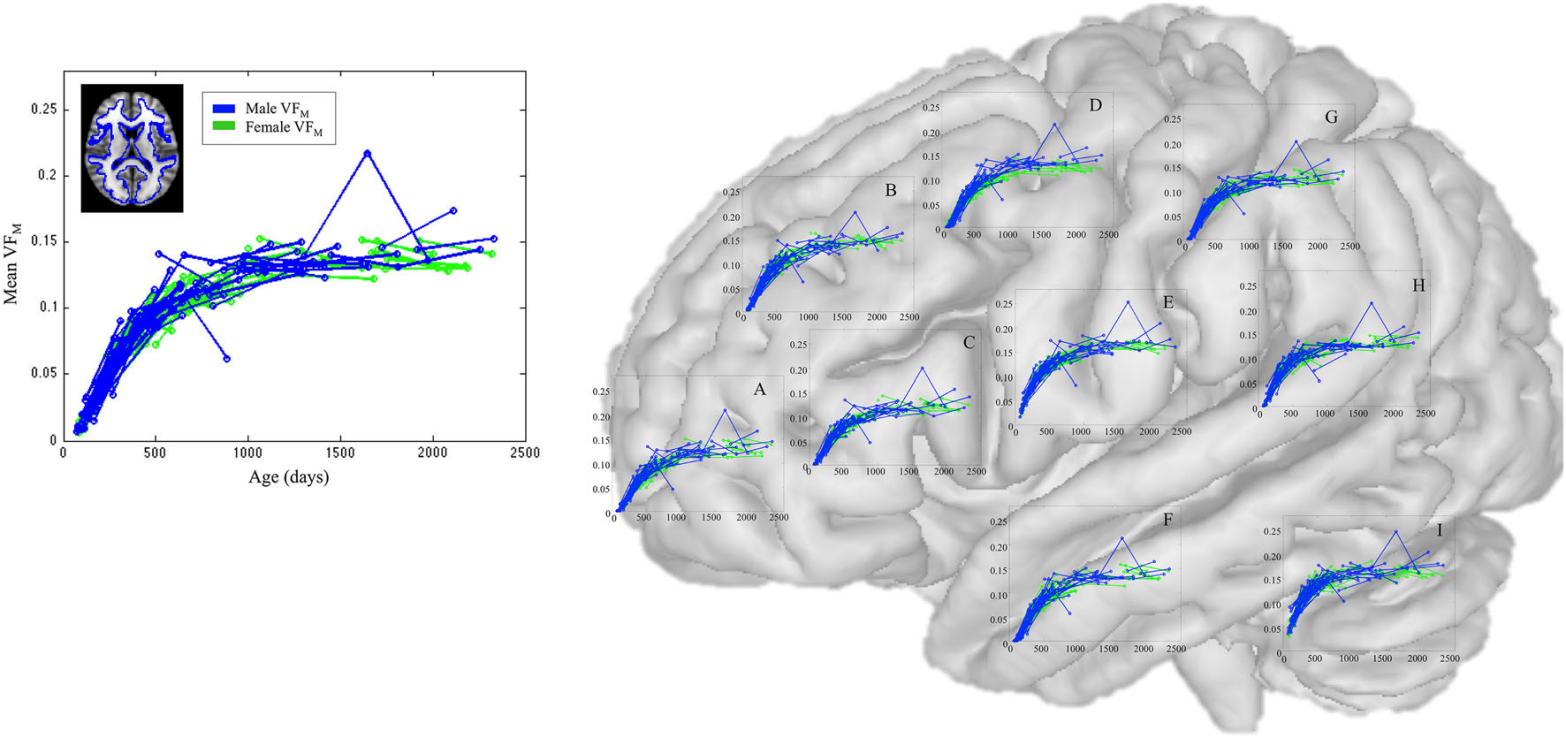
Representative mean VF_M_ developmental trajectories. Repeated measurements for each subject are connected with a straight line. Developmental trajectories are observed to follow a “S” shape pattern that is characteristic to a sigmoidal function. Anatomical locations associated with each graph: *A* frontal lobe white matter, *B* caudate, *C* insula, *D* putamen, *E* thalamus, *F* temporal lobe white matter, *G* parietal lobe white matter, *H* occipital lobe white matter, *I* cerebellar white matter

Longitudinal VF_M_ developmental trajectories for each of the 28 investigated regions (Supplementary Fig. 2) are shown in Fig. 2. Subject-specific measurements are connected with a straight line between each repeated time point. VF_M_ increases nonlinearly with age, following a characteristic sigmoidal pattern with more rapid changes at early ages and slower development at older ages. This temporal pattern, beginning in deep and progressing to superficial white matter in a caudal–rostral direction, qualitatively agrees with previously described histological trajectories (Flechsig 1901; Yakovlev and Lecours 1967). It is noteworthy that although regional developmental trajectories were similar in profile, distinct temporal patterns are observed for specific regions. For example, frontal white matter is observed to have a longer lag in development compared to occipital and parietal white matter regions, which is in agreement with the frontal lobes being a late developing brain region.

### Nonlinear mixed modeling of developmental trajectories

**Fig. 3:**
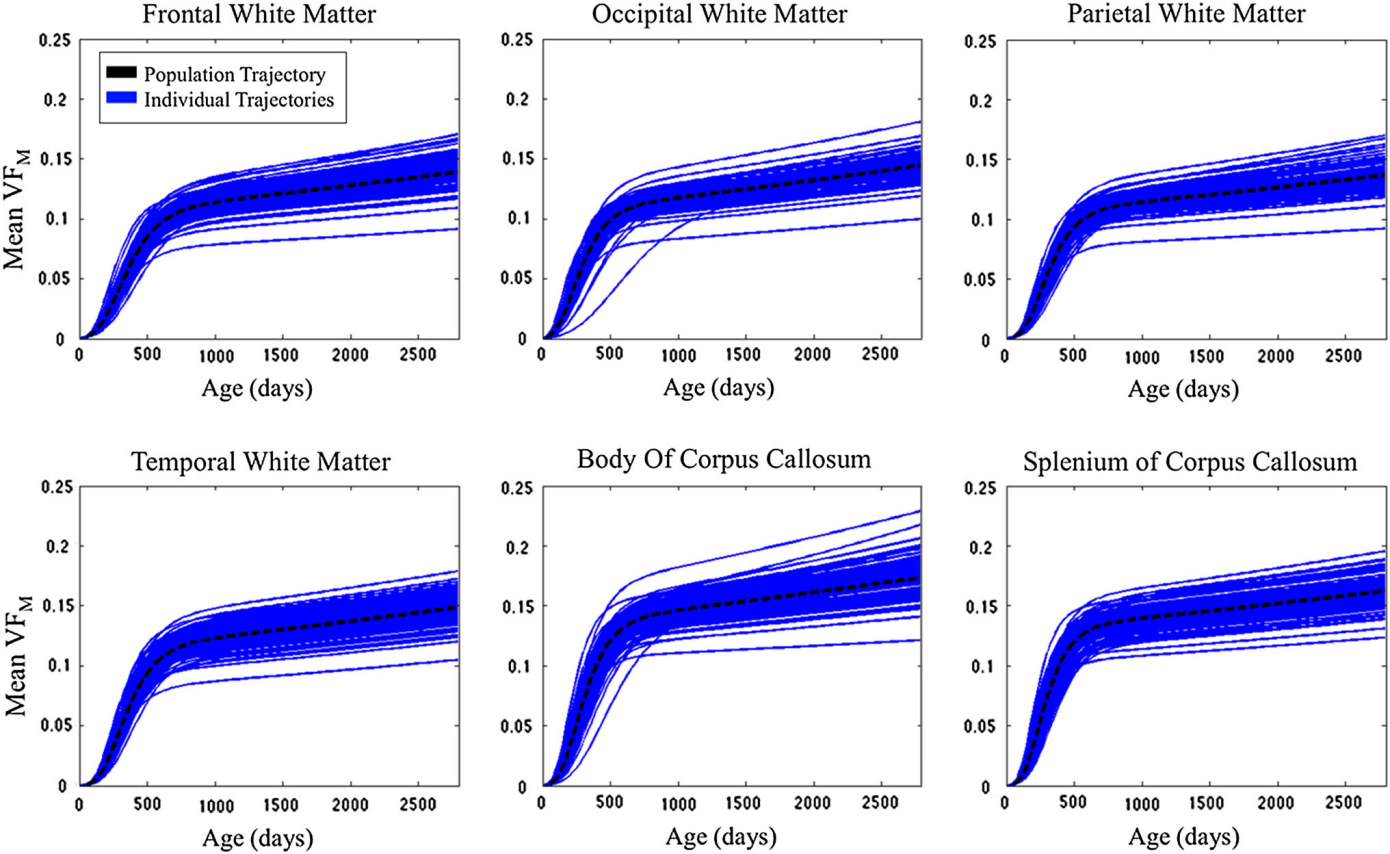
Reconstructed modified Gompertz trajectories for each subject (*blue curves*) and the overall population (*black dashed curve*) for a representative subset of the investigated regions. Parameters for these models were estimated by fitting the VF_M_ profile to the modified Gompertz function as a function of gestationally corrected age using nonlinear mixed effects modeling

**Fig. 4:**
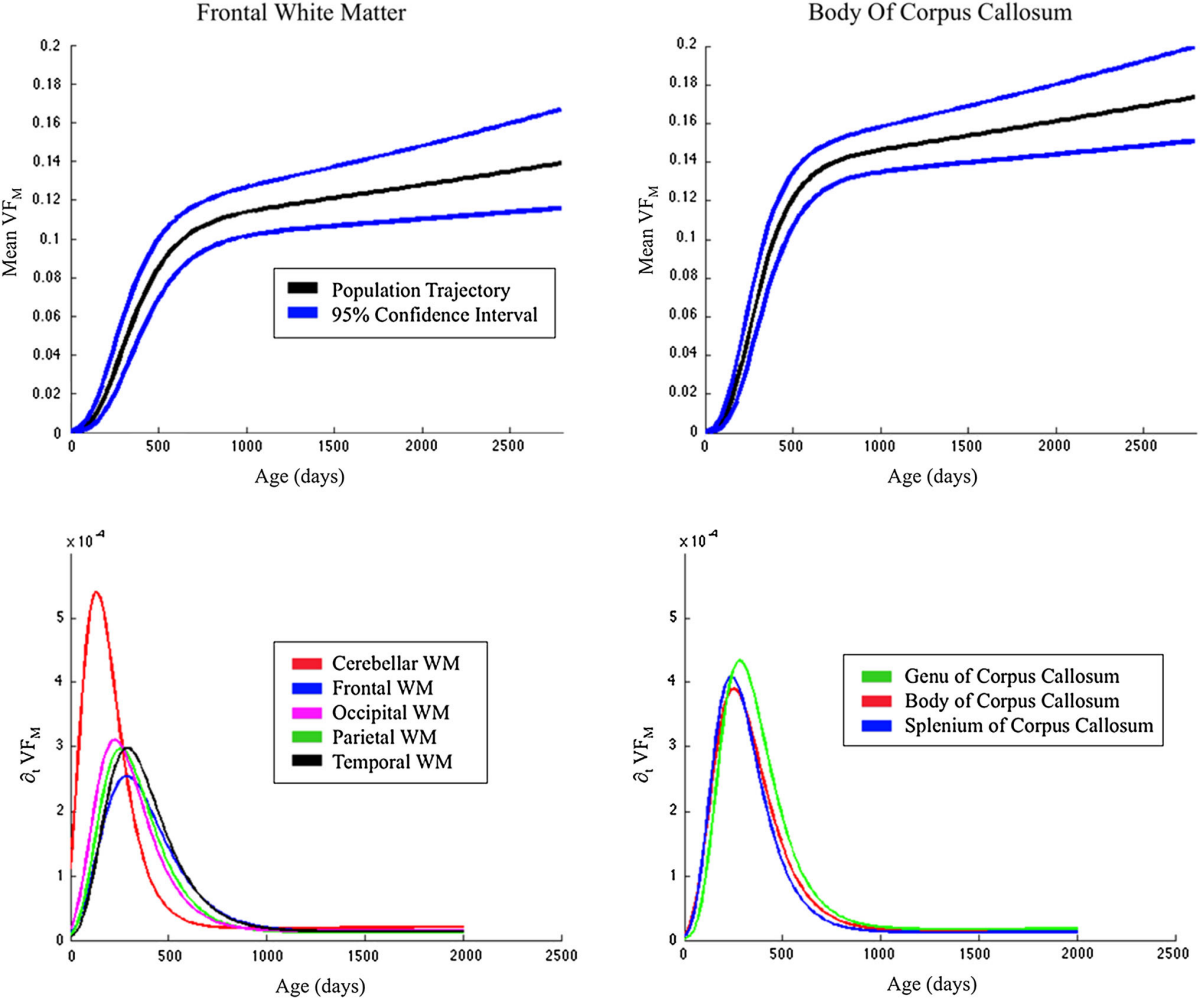
*Top* *Row*: 95 % confidence intervals for the frontal white matter and body of the corpus callosum. Using the standard error estimates from the nonlinear mixed effects modeling, confidence intervals of the population trajectory can be estimated. *Bottom row*: representative VF_M_ growth rate curves. These curves were reconstructed by taking the time derivative of the modified Gompertz function (Eq. 2) and using the overall population estimates of the modified Gompertz parameters. Such curves are informative of the rate of change of the VF_M_ with respect to age (time) and highlight the posterior–anterior developmental gradient of VF_M_

The mixed effect modeling framework was applied to the 28 regional VF_M_ trajectories to obtain Gompertz growth curve parameter estimates of both population fixed and subject-specific random effects. Representative modeled developmental trajectories are shown in Fig. 3, with overall population curves overlaid on top of subject-specific developmental trajectories. Additional trajectories are provided in Supplementary Figure 4. These plots illustrate both the degree of individual variability of VF_M_ development, as well as the ability of the modeling framework to capture the population’s overall growth pattern. Moreover, mixed effects modeling provides standard error estimates of the fixed parameters, allowing derivation of confidence intervals of the developmental trajectory (Fig. 4).

Using the described population Gompertz parameter estimates, curves of the VF_M_ growth rate were also derived; indicating how fast VF_M_ changes over time. The rate of myelination is most intense at earlier ages for core white matter, whereas the fastest rate of myelin development occurs later in peripheral cortical regions (Fig. 4).

### Gender differences

**Fig. 5:**
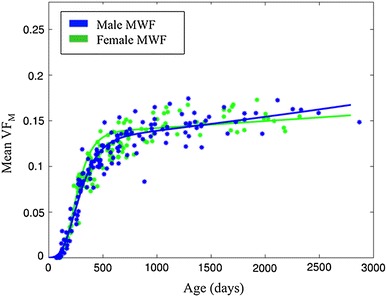
VF_M_ developmental trajectory of the splenium of the corpus callosum. Trajectories are separated by gender, highlighting the developmental profile difference between males and females

Results from comparing the nonlinear mixed effect models fitting of gender-separated data and the male/female pairwise *t* tests between estimated Gompertz parameters are shown in Supplementary Table 4. *F*-statistics revealed no differences in the developmental trajectory residuals between the gender-separated and combined data. However, pairwise differences between male and female fixed-effect Gompertz parameter estimates were observed to be significant (Supplementary Table 4). For example, males were found to have significantly larger *α* values in the splenium of the corpus callosum, corresponding to an increased maximal VF_M_ value; while differences in other model parameters (development lag, *β*; initial development rate, *γ*; and secondary development rate, *δ*) were found to vary between males and females in a majority of the regions investigated. On average, males were found to have a significantly larger secondary developmental rate (*δ*) than females, while females were found to have a larger increased maximal VF_M_ (*α*) than males. Differences between these parameters are reflected in the male and female developmental trajectories, as shown in Fig. 5 for the splenium of the corpus callosum.

### Developmental relationships with cognitive scores

**Fig. 6:**
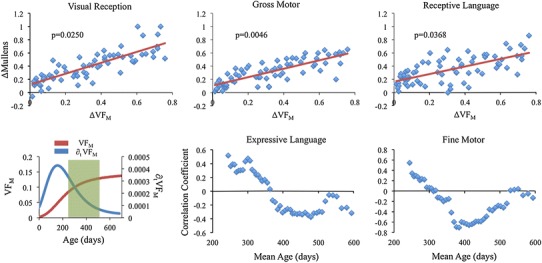
*Top row*: change in visual reception, gross motor, and receptive language Mullen scales of early learning assessment scores as a function of changing VF_M_ for the thalamus. The change in cognitive assessment scores were found to significantly correlate with changing VF_M_, suggesting concomitant structure–function development. *Bottom row*: illustrative moving average correlations (Pearson’s r, *y*-axis) as a function of mean age (days, *x*-axis) for the thalamus. Plots demonstrate the dynamic relationships between $\varDelta_{{{\text{VF}}_{\text{M}} }}$ and fine motor and expressive language Δ_M_. The age range at which these relationships transition appears to overlap with changes in the VF_M_ and ∂_t_ VF_M_ trajectories

Longitudinal changes VF_M_ were found to significantly (*p* < 0.05, corrected for multiple comparisons) correlate with changes in Mullen assessment scores. Representative plots illustrating the positive relationship between the change in VF_M_ and the change in Mullen assessment scores are shown in the top row of Fig. 6 for the thalamus. Gross motor scores were correlated with VF_M_ in areas of the basal ganglia, thalamus, cerebellum, and other core white matter tracts, while measures of visual reception were correlated with VF_M_ in the posterior limb of the internal capsule, superior corona radiata, and the superior longitudinal fasciculus. Changes in receptive language scores were also correlated with changes in VF_M_ in the cerebellum, thalamus, occipital white matter, and posterior thalamic radiations.

While no correlations were found to be significant between changes in expressive language or fine motor, the relationship between $\varDelta_{{{\text{VF}}_{\text{M}} }}$ and Δ_M_ for these scores was found to change over time. This age interaction is illustrated using a moving bin correlation in the bottom row of Fig. 6 for the thalamus. Prior to 350 days and following approximately 500 days, the correlation between expressive language Δ_M_ and $\varDelta_{{{\text{VF}}_{\text{M}} }}$ thalamus became increasingly negative, while in between this age range, the relationship appeared to become constant. The correlation between fine motor Δ_M_ and $\varDelta_{{{\text{VF}}_{\text{M}} }}$ thalamus became increasingly negative until approximately 500 days, before becoming increasingly positive. Similar age interactions with expressive language were observed in the posterior limb of the internal capsule and cerebellar white matter, while fine motor scores were detected to change over time in left hemispheric posterior limb of the internal capsule. A complete summary of the results from the general linear model analysis is provided in Supplementary Table 5.

## Discussion

In this work, we have investigated longitudinal brain development from infancy through to early childhood using quantitative multicomponent relaxometry for the first time. From a large cohort of 108 typically developing male and female children, we have shown that regional VF_M_ trajectories follow a nonlinear growth pattern, consistent with the myelination patterns of both histological studies (Flechsig 1901; Yakovlev and Lecours 1967) and our own prior cross-sectional VF_M_ imaging studies of white matter development (Deoni et al. 2012). Using nonlinear mixed effects modeling, we have derived population and subject-specific growth model parameters that characterize underlying VF_M_ development and may be used to reconstruct normative reference growth and growth rate curves (as illustrated in Figs. 3, 4, 5). We have further examined the relationships between VF_M_ maturation and cognitive development. Our results show a strong correlation between changes in VF_M_ and scales of visual reception, gross motor, and receptive language, while the relationships between VF_M_ and expressive language and fine motor were found to change in time. These findings support previous reports of the concomitant emergence of myelinated white matter and cognitive function and more importantly, may reveal a window into the timing of biological changes that shape behavioral development.

Characterization of longitudinal changes of myelin content is particularly well suited to examine the spatio-temporal relationships of brain development. Myelination undergoes a dramatic increase during the first years of life in response to activity-based learning and plasticity, with the greatest rates of change occurring before 3 years of age (Yakovlev and Lecours 1967). Tracking these nonlinear changes using quantitative MRI techniques sensitive to myelination allows us to examine this neurodevelopmental process in vivo and quantitatively map the myelination trajectory (Deoni et al. 2011, 2012). Establishment of VF_M_ growth and rate of growth trajectories offers the potential to distinguish typical and atypical myelination patterns, as well as determine the age at which such deviations from the normal trajectory occur (Dean et al. 2014a, b). This is increasingly important to the study of neurobehavioral and neuropsychiatric disorders, as many of the pathological features of these diseases are consistent with disrupted or aberrant myelination (Fields 2008).

Modeling of the nonlinear white matter maturation provides a more detailed examination of the microstructural changes that take place during neurodevelopment. Such analysis provides a unique parameterization of the developmental trajectory, yielding intuitive growth parameters related to the underlying development (Sadeghi et al. 2013). Each of these parameters has a distinctive role on the overall curve (Supplementary Fig. S3), and therefore, interrogation of these parameters may be useful in future investigations. Specifically, the modified Gompertz growth function (Eq. 1) applied throughout this work enables the trajectory to be put in terms of amplitude (*α*), lag (*β*), and growth rates (γ, *δ*). While the use of a three-parameter Gompertz function $\left( {f(t) = \alpha \,\exp \,( - \beta \,\exp ( - \gamma_{\text{t}} ))} \right)$ has previously been used to characterize the development of DT-MRI measures, including FA, MD, and RD, in young children (Sadeghi et al. 2013) the function described in these works are limited to describing continued developmental changes observed to take place in adolescence and adulthood (Lebel and Beaulieu 2011) due to the asymptotic nature of the function. Due to the additional model parameter, the modified Gompertz function allows for continued growth and has been shown to outperform other growth models, including the three-parameter Gompertz function, at characterizing observed patterns of cross-sectional VF_M_ development (Dean et al. 2014a). Nonetheless, while these two mathematically similar models reflect the development of different MRI metrics, comparison of the Gompertz parameters from these separate but complementary models may be interesting to examine the relationships between DT-MRI and MCR metrics in neurodevelopment.

In addition to comparing growth models constructed from differing imaging techniques, a more complete characterization of underlying microstructural white matter and myelin development may be realized by integrating these distinct quantitative imaging methods into a single neuroimaging protocol. Development of such a protocol would require optimization of the scan pulse sequences for children (Deoni et al. 2012) and scan acquisition times would need to be considered, as longer scans can make acquiring artifact-free MR images from children more challenging (Dean et al. 2014b). Though we are unaware of any current study that incorporates this multimodal information, combining MCR with DT-MRI, MT-imaging measures, as well as metrics of cortical thickness (CT) and functional brain activity (fMRI and/or EEG) would be informative to the study of brain development. Such a study would allow the relationships between these separate measures to be investigated as well as provide a more thorough description of the structural and functional changes that take place as the brain matures.

In addition, mixed effects modeling of longitudinal data is advantageous to other cross-sectional modeling techniques at characterizing brain development due to its ability to provide information that is relevant to population and individual growth. Mixed effects methods provide a more flexible modeling framework, as repeated measurements from the same individual are easily handled and missing observations from an individual do not require the entire set of individual measurements to be removed from the analysis. The success of these types of mixed model designs has been demonstrated in studies examining brain development in young children (Sadeghi et al. 2013) and adolescents (Lebel and Beaulieu 2011). Our results complement these previous studies to provide the first mixed effects analysis of VF_M_ development, allowing for the creation of population and individual VF_M_ developmental trajectories for the first time. Establishment of such individual VF_M_ growth trajectories may provide the ability to pinpoint subtle individual variations of white matter development. This feature of mixed effects models is essential for their continued use in neuroimaging and identifying specific characteristics within individuals who go on to develop neurodevelopmental disorders (Xu et al. 2008).

Developmental trajectories are often reported to differ between sexes. Lenroot et al. (2007) reported distinguishable longitudinal patterns of brain development between males and females, observing males to have accelerated white matter development when compared to females. Surprisingly, comparison of the overall trajectories between males and females yielded no significant differences in any of the examined regions. Such findings may be a result of the region of interest-type of analysis performed, in which subtle trajectory differences between males and females were masked by averaging large numbers of voxels. Extension of the regional analysis to a voxelwise inspection may be more informative and unmask subtle sex differences. Nevertheless, pairwise comparison of the modified Gompertz modeling parameters revealed significant differences in the majority of regions (Supplementary Table 4). These differences may be interpreted in terms of the defining characteristics that each parameter represents in the modified Gompertz model. For example, the parameters *γ* and *δ* represent VF_M_ growth rates in the modified Gompertz model and, therefore, our analysis suggests that the majority of VF_M_ growth rates were significantly higher in males than females. Females were found to have significantly larger *α* values in most of the examined regions, suggesting the amplitude of the developmental trajectory to be larger in females. These results are consistent with previous studies that have reported males to have steeper rates of increase of white matter volume (Lenroot et al. 2007) while females have been observed to have higher FA values relative to males in adolescents (Bava et al. 2008). While the cause for these sex differences is unknown, it has been hypothesized elsewhere that varying exposure levels to sex hormones during development may influence the maturational trajectory of the brain (Neufang et al. 2009). Other extraneous factors, including socioeconomics and genetic makeup, are also likely to affect the developmental trajectory of the brain (Giedd and Rapoport 2010) and thus further examination of the role of these characteristics on sexual dimorphic neurodevelopment is essential.

Myelination of white matter pathways is an integral component to the development of cognitive function during childhood, as it allows for faster electrical conduction and therefore more efficient communication in the brain. Based on the results from previous neuroimaging studies (Dubois et al. 2008; Short et al. 2013), we anticipated on finding relationships between longitudinal measures of VF_M_ and Mullen assessment measures. Our results show longitudinal changes of VF_M_ to positively correlate with changes in gross motor, receptive language, and visual reception, while the correlation between VF_M_ and scales of expressive language and fine motor was found to change with age. These results are suggestive that individual differences in the rate of VF_M_ change over time are associated with individual differences in the rate of behavioral change over time. Moreover, the relationships between these structural and functional changes are dynamically influenced by age (as illustrated in Fig. 6). Such a result alludes to the time interval between 1 and 2 years of age as representing a transitionary period of brain maturation, a particularly important stage of development that has been previously been indicated as a time in which neurodevelopmental disorders, such as autism, manifest (Courchesne et al. 2007; Bale et al. 2010). These results are additionally consistent with the interpretations of previous works in that there exists an interdependent relationship between cognitive and behavioral functions and the myelination of inter-connected brain networks that depend on the rapid transmission of information (Casey et al. 2005; Fields 2008; Tamnes et al. 2010). However, while this interpretation seems plausible given our observations, studies investigating the extrinsic and intrinsic factors that influence myelination are needed to further examine this hypothesis.

Of the five Mullen scales, changes in gross motor ability correlated with changes in VF_M_ in the most number of regions (22 of 28). Developing gross motor ability is central to the development of skills and abilities that are related to large movements (i.e., crawling, walking, head control, posture) and also play a primary role in the other four cognitive domains (Mullen 1995). These abilities require a foundation of evolved communication pathways and may be the reason why this measure is observed to be correlated with the numerous brain regions. Other positive correlations found between the changes in visual reception and VF_M_ involved regions (thalamus, internal capsule, corona radiata) associated with the central visual pathways, signifying the myelination of these areas lead to improved visual processing skills. Likewise, regions correlated with changes in receptive language, including cerebellar white matter, thalamus, posterior internal capsule, superior longitudinal fasciculus, have previously been identified to be associated with changes in language comprehension (Geschwind 1970; Bernal and Altman 2010). Although these results are preliminary, our data lend itself to further investigation of these relationships. In particular, examining the ability of maturing white matter to predict subsequent functional connectivity could prove to be informative in understanding how variations of the structural properties of white matter systematically influence cognitive function. Moreover, as the integration of functional resting state data and/or EEG data with quantitative MRI measurements has recently been successful at examining the functional maturation of white matter (Dubois et al. 2008), incorporating these additional functionally relevant data with VF_M_ measurements would likely be beneficial to understanding the structure–function relationships of the developing brain.

Though mcDESPOT is a recent MCR imaging technique that affords rapid, whole-brain coverage, the imaging technique differs from conventional and established multi-spin echo methods (Zhang et al. 2014). In particular, conventional VF_M_ derived from multi-echo spin echo measurements has shown to strongly correlate to histological measurements of myelin content (Laule et al. 2008) and provide a more specific marker for myelin than parameters derived from diffusion tensor (Mädler et al. 2008) and magnetization transfer imaging (Vavasour et al. 1998). Histological comparisons of mcDESPOT VF_M_ measures have shown strong qualitative agreement in the Shaking Pup model of dysmyelination (Hurley et al. 2010), though quantitative comparisons remain to be carried out. Prior mcDESPOT studies of neurodevelopment have reproduced the known histologically established spatial–temporal pattern of myelination, and VF_M_ measures have been shown to reflect clinical disability in known demyelinating disorders, including multiple sclerosis (Kitzler et al. 2012; Kolind et al. 2012) and amyotrophic lateral sclerosis (Kolind et al. 2013). Such comparisons give confidence that if mcDESPOT VF_M_ measures are not uniquely specific to myelin, they provide unique information that is strongly sensitive to myelin content. Still, additional studies examining the specificity of mcDESPOT-derived VF_M_ measures to myelin, including quantitative comparison to histological stains of myelin content, are necessary to validate the mcDESPOT technique as a viable “myelin imaging” technique. These studies are of great interest and will be beneficial as an area of future research.

The region of interest-based analyses performed in this work are useful for characterizing the average developmental trajectory of specific, anatomically defined brain regions; however, voxel-based methods may be more revealing as these methods provide information across the whole brain. Performing the mixed effects modeling of our longitudinal data at the voxel level would provide estimates of the overall population (fixed effects) and subject-specific (random effects) modified Gompertz parameters at each imaging voxel, allowing us to predict VF_M_ values at later ages across the whole brain. This is important for establishing a normative model of neurodevelopment and having the ability to identify deviations from typical growth as these differences are unlikely to be restricted to grossly defined regions of interest (Snook et al. 2007). For this reason, future work extending the nonlinear mixed effects modeling to cover the whole brain is planned.

In summary, we have presented the first longitudinal examination of white matter development throughout early childhood. The results reported from previous cross-sectional studies of VF_M_ maturation are extended by showing longitudinally that VF_M_ follows a specific, nonlinear trajectory throughout early childhood and is qualitatively consistent with spatial–temporal patterns of myelination of the human brain. We have shown that VF_M_ trajectories of the normative population and individual subjects can be characterized and predicted by modeling for the nonlinear growth patterns using a nonlinear mixed effects framework. Moreover, these VF_M_ developmental trajectories differed between males and females, while changes in VF_M_ were significantly correlated with changing measures of cognition and behavior. The coupling between this structural and functional development provides insight into the evolving relationship between myelin content and behavior as well as indicating evidence of sensitive periods of neurodevelopment between 1 and 2 years of age. This presented work provides an important step for understanding the typical patterns of normative white matter maturation and its relationship to emerging cognition as well as providing a foundation for future studies examining aberrant brain development.

## Electronic supplementary material

Below is the link to the electronic supplementary material.

Supplementary Figure 1: Regions of interest and white matter tracts used for regional analysis. Abbreviations are as follows: SLF: superior longitudinal fasciculus; ACR: anterior corona radiata; SCR: superior corona radiata; PTR: posterior corona radiata; ALIC: anterior limb of the internal capsule; PLIC: posterior limb of the internal capsule; CC: corpus callosum; PCR: posterior corona radiata (TIFF 543 kb)

Supplementary Figure 2: Illustration of the longitudinal registration pipeline (TIFF 466 kb)

Supplementary Figure 3: Effect of varying parameter values on the modified Gompertz function. The red curve is fixed throughout the 4 different scenarios, while the values of the varied parameter are given in each plot legend (TIFF 176 kb)

Supplementary Figure 4: Modeled developmental trajectories from the remaining 22 regions of interest (TIFF 472 kb)

Supplementary material 5 (DOCX 106 kb)

Supplementary material 6 (DOCX 68 kb)

Supplementary material 7 (DOCX 76 kb)

Supplementary material 8 (DOCX 106 kb)

Supplementary material 9 (DOCX 133 kb)
