# Supplementary figures and images for: Characterizing longitudinal white matter development during early childhood
Source: Brain Struct Funct. 2014 Apr 8;220(4):1921–33. doi: 10.1007/s00429-014-0763-3 (PMC4481335; doi:10.1007/s00429-014-0763-3)

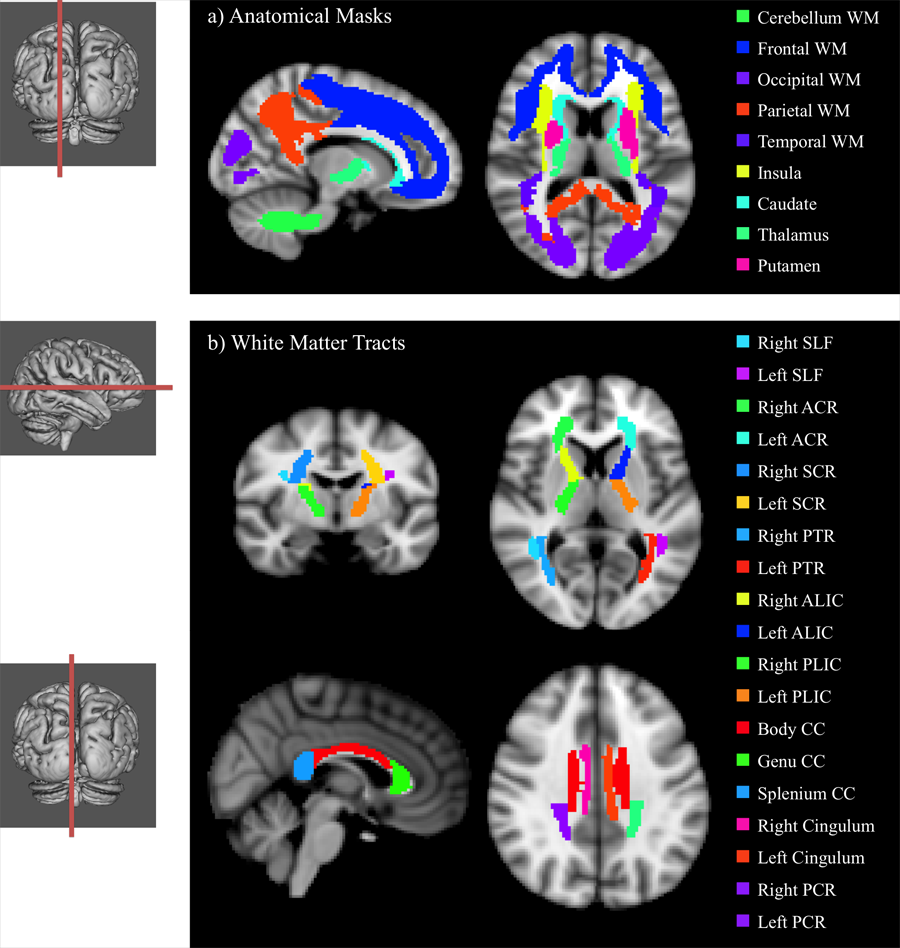

Supplement: Supplementary file 1 — Supplementary Figure 1: Regions of interest and white matter tracts used for regional analysis. Abbreviations are as follows: SLF: superior longitudinal fasciculus; ACR: anterior corona radiata; SCR: superior corona radiata; PTR: posterior corona radiata; ALIC: anterior limb of the internal capsule; PLIC: posterior limb of the internal capsule; CC: corpus callosum; PCR: posterior corona radiata (TIFF 543 kb) [file 429_2014_763_MOESM1_ESM.tif]

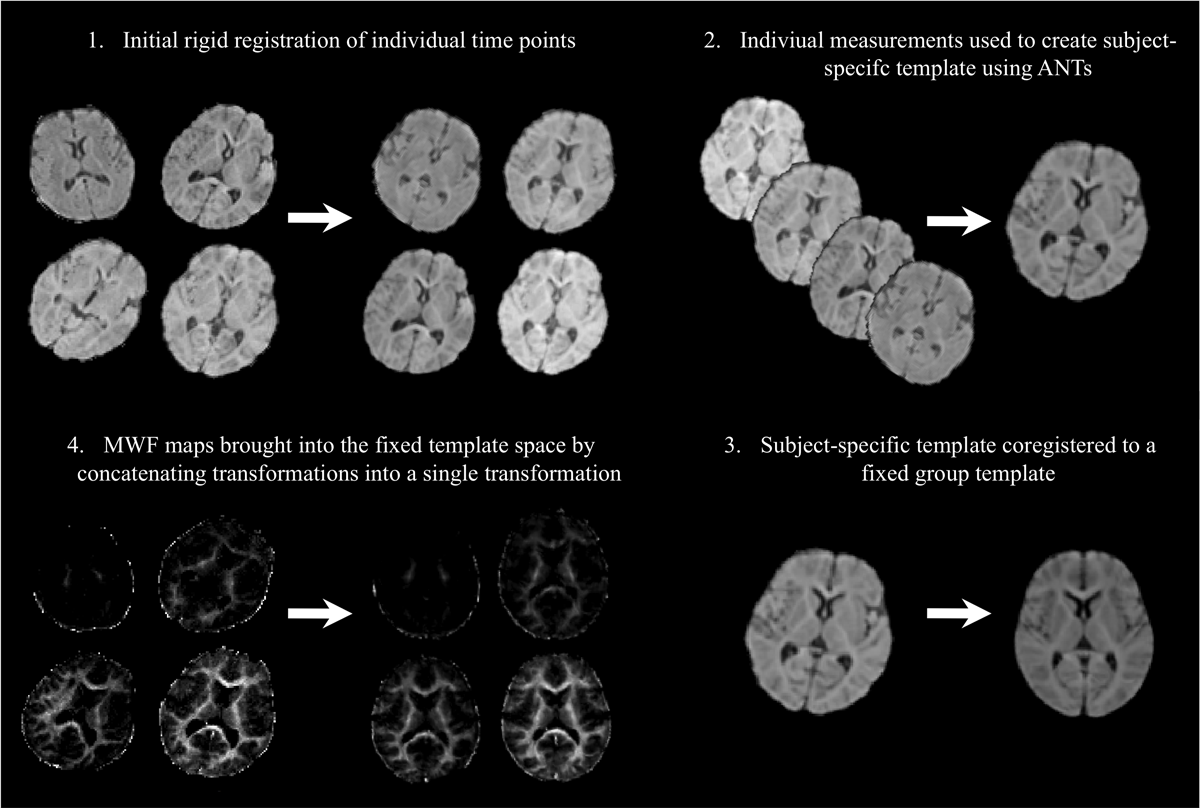

Supplement: Supplementary file 2 — Supplementary Figure 2: Illustration of the longitudinal registration pipeline (TIFF 466 kb) [file 429_2014_763_MOESM2_ESM.tif]

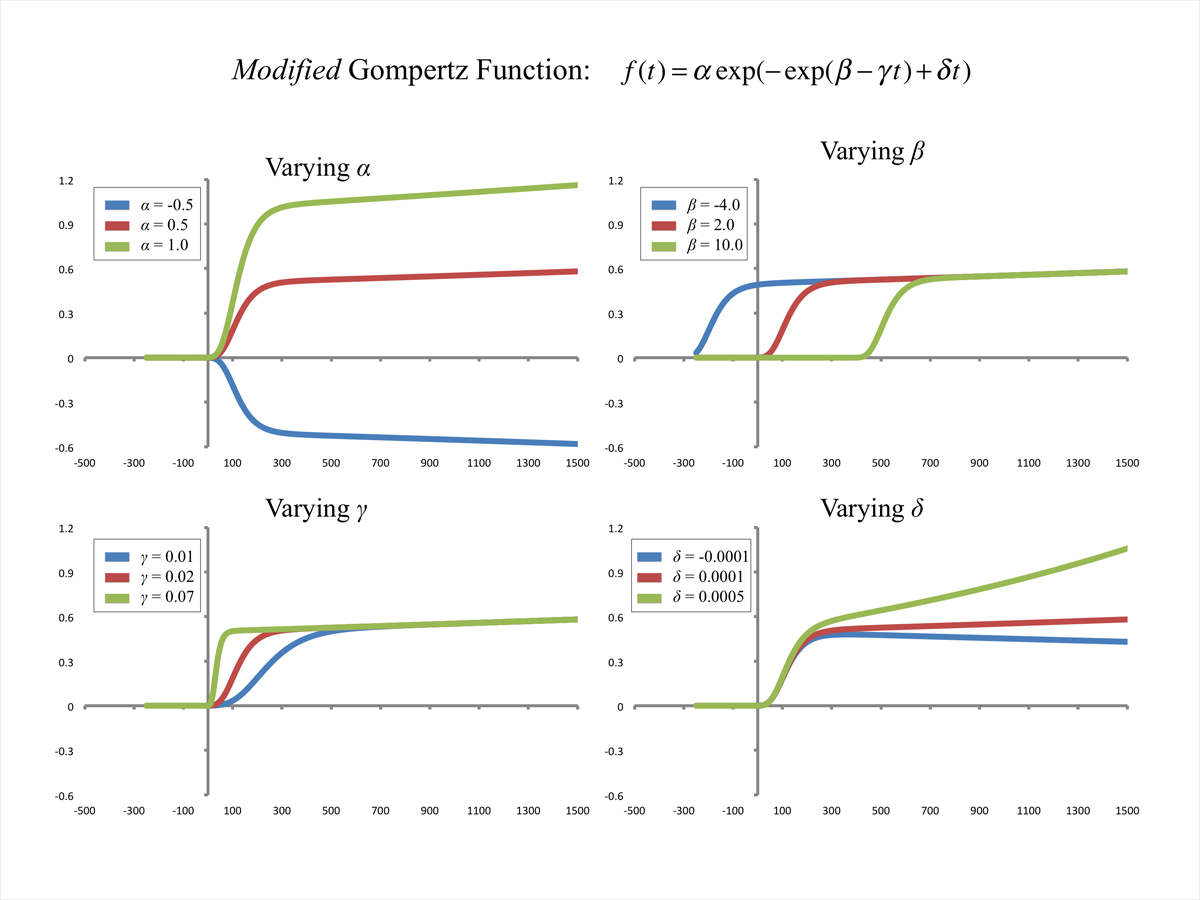

Supplement: Supplementary file 3 — Supplementary Figure 3: Effect of varying parameter values on the modified Gompertz function. The red curve is fixed throughout the 4 different scenarios, while the values of the varied parameter are given in each plot legend (TIFF 176 kb) [file 429_2014_763_MOESM3_ESM.tif]

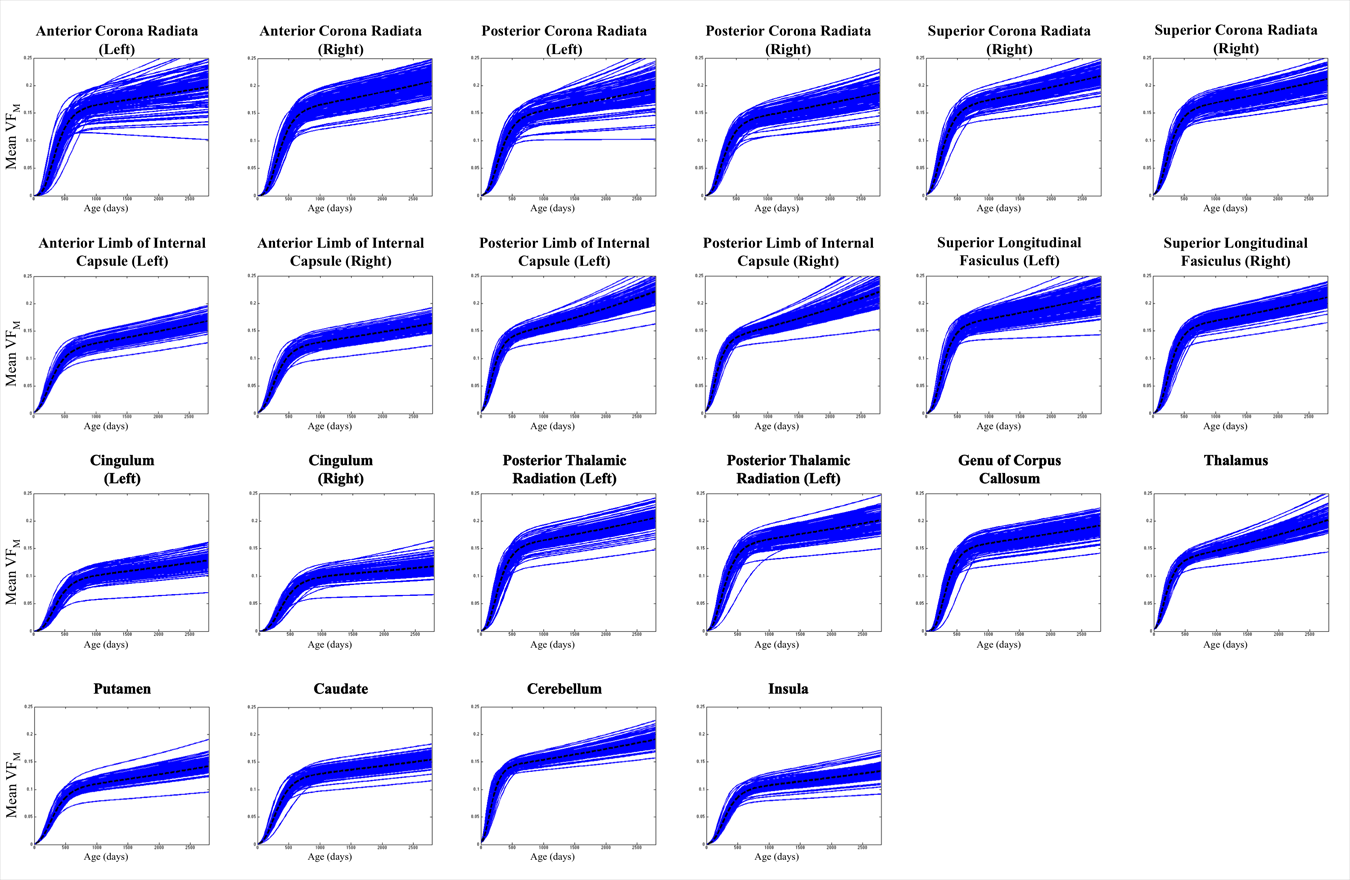

Supplement: Supplementary file 4 — Supplementary Figure 4: Modeled developmental trajectories from the remaining 22 regions of interest (TIFF 472 kb) [file 429_2014_763_MOESM4_ESM.tif]
