# Supplementary material for: Characterizing longitudinal white matter development during early childhood
Source: Brain Struct Funct. 2014 Apr 8;220(4):1921–33. doi: 10.1007/s00429-014-0763-3 (PMC4481335; doi:10.1007/s00429-014-0763-3)
Supplement: Supplementary file 5 — Supplementary material 5 (DOCX 106 kb) [file 429_2014_763_MOESM5_ESM.docx]

**Supplementary Table 1:** Child demographic information of 108 participants divided by age group. Age information reflects the age of the participants when first enrolled in the study.

|  | **3 months** | **6 months** | **9 months** | **12 months** | **15 months** | **18 months** | **21 months** |
| --- | --- | --- | --- | --- | --- | --- | --- |
| Participants | 22 | 19 | 9 | 12 | 6 | 3 | 7 |
| Male / Female | 14 / 8 | 10 / 9 | 6 / 3 | 2 / 10 | 4 / 2 | 1 / 2 | 2 / 5 |
| Age Range (days) | 70 - 129 | 140 - 201 | 231 - 309 | 326 - 403 | 413 - 489 | 500 - 575 | 594 - 689 |
| Mean ± Std. Age | 103.86 ± 17.07 | 180.00 ± 23.11 | 264.44 ± 24.10 | 365.83 ± 21.29 | 465.00 ± 30.33 | 530.33 ± 39.50 | 652.00 ± 31.33 |
| Gestational Period (weeks) | 39.57 ± 1.29 | 39.26 ± 1.09 | 39.25 ± 1.43 | 39.75 ± 1.13 | 39.29 ± 2.25 | 39.67 ± 1.95 | 38.39 ± 1.05 |
| Birth Weight (oz) | 122.20 ± 15.92 | 117.21 ± 15.84 | 132.11 ± 19.07 | 121.67 ± 11.83 | 116.17 ± 7.83 | 130.33 ± 11.93 | 118.43 ± 15.69 |
| Birth Height (in) | 20.22 ± 1.25 | 19.82 ± 1.15 | 21.27 ± 0.64 | 20.36 ± 0.67 | 19.80 ± 0.65 | 20.33 ± 0.58 | 19.82 ± 1.36 |
| Ethnicity [Hispanic / Non-Hispanic / Not Reported] | 2 / 18 / 2 | 4 / 14 / 1 | 0 / 9 / 0 | 2 / 9 / 1 | 0 / 5 / 1 | 0 / 3 / 0 | 1 / 6 / 0 |
| Race [African American / Asian/ Caucasian / Mixed Race / Unknown or Not Reported] | 3 / 0 / 15 / 2 / 2 | 0 / 0 / 14 / 4 / 1 | 2 / 0 / 7 / 0 / 0 | 2 / 0 / 7 / 1 / 2 | 0 / 0 / 4 / 1 / 1 | 0 / 0 / 2 / 1 / 0 | 1 / 0 / 5 / 1 / 0 |
|  |  |  |  |  |  |  |  |
|  | **24 months** | **30 months** | **36 months** | **42 months** | **48 months** | **54 months** | **60 months** |
| Participants | 3 | 8 | 4 | 3 | 4 | 4 | 4 |
| Male / Female | 2 / 1 | 8 / 0 | 2 / 2 | 2 / 1 | 2 / 2 | 0 / 4 | 3 / 2 |
| Age Range | 719 - 752 | 791 - 981 | 1052 - 1094 | 1210 - 1304 | 1368 - 1451 | 1618 - 1705 | 1728 - 1924 |
| Mean ± Std. Age | 731.67 ± 17.79 | 898.25 ± 89.55 | 1067.00 ± 18.78 | 1248.00 ± 49.52 | 1406.75 ± 34.28 | 1671.25 ± 38.74 | 1829.50 ± 103.89 |
| Gestational Period (weeks) | 38.57 ± 0.87 | 39.41 ± 1.11 | 39.57 ± 1.71 | 40.38 ± 0.58 | 39.46 ± 1.89 | 39.82 ± 1.73 | 38.79 ± 0.94 |
| Birth Weight (oz) | 125.33 ± 9.02 | 118.25 ± 20.18 | 119.75 ± 16.40 | 102.33 ± 17.16 | 116.25 ± 14.38 | 128.50 ± 20.49 | 112.25 ± 10.01 |
| Birth Height (in) | 30.33 ± 1.15 | 20.13 ± 1.22 | 20.67 ± 2.08 | 20.42 ± 0.63 | 17.95 ± 1.48 | 20.83 ± 0.29 | 21.83 ± 3.82 |
| Ethnicity [Hispanic / Non-Hispanic / Not Reported] | 0 / 3 / 0 | 1 / 7 | 2 / 2 / 0 | 2 / 1 / 0 | 1 / 3 / 0 | 3 / 1 / 0 | 1 / 3 / 0 |
| Race [African American / Asian/ Caucasian / Mixed Race / Unknown or Not Reported] | 0 / 0 / 3 / 0 / 0 | 1 / 1 / 5 / 1 / 0 | 1 / 0 / 2 / 0 / 1 | 1 / 0 / 1 / 0 / 1 | 0 / 0 / 3 / 0 / 0 | 0 / 0 / 2 / 2 / 0 | 0 / 0 / 4 / 0 / 0 |
