# Supplementary material for: Characterizing longitudinal white matter development during early childhood
Source: Brain Struct Funct. 2014 Apr 8;220(4):1921–33. doi: 10.1007/s00429-014-0763-3 (PMC4481335; doi:10.1007/s00429-014-0763-3)
Supplement: Supplementary file 6 — Supplementary material 6 (DOCX 68 kb) [file 429_2014_763_MOESM6_ESM.docx]

**Supplementary Table 2:** mcDESPOT imaging protocols optimized for young children

| **Age Group (months)** | **3-9** | **9-16** | **16-28** | **28-48** | **48+** |
| --- | --- | --- | --- | --- | --- |
| **Acquisition Time (min:sec)** | 18:22 | 18:42 | 21:38 | 24:20 | 22:45 |
| **Field of View (cm^3^)** | 14 x 14 x 13 | 17 x 17 x 14.4 | 18 x 18 x 15 | 20 x 20 x 15 | 20x20x16.5 |
| **SPGR TR / TE (ms)** | 12 / 5.8 | 12 / 5.9 | 12 / 5.4 | 11 / 5.2 | 10 / 4.8 |
| **SPGR Flip Angles (degrees)** | 2, 3, 4, 5, 7, 9, 11, 14 | 2, 3, 4, 5, 7, 9, 11, 14 | 2, 3, 4, 5, 7, 9, 11, 14 | 2, 3, 4, 5, 7, 9, 12, 16 | 3, 4, 5, 6, 7, 9, 13, 18 |
| **IR-SPGR Inversion Time (ms)** | 600 / 950 | 600 / 900 | 500 / 850 | 500 / 800 | 450 / 750 |
| **bSSFP TR / TE (ms)** | 10 / 5 | 10.2 / 5.1 | 10 / 5 | 9.8 / 4.4 | 10 / 4.8 |
| **bSSFP Flip Angles (degrees)** | 9, 14, 20, 27, 34, 41, 56, 70 | 9, 14, 20, 27, 34, 41, 56, 70 | 9, 14, 20, 27, 34, 41, 56, 70 | 9, 14, 20, 27, 34, 41, 56, 70 | 9, 14, 20, 27, 34, 41, 56, 70 |
| **Unprotected dB*** | 54 | 62 | 69 | 74 | 82 |
