# Supplementary material for: Characterizing longitudinal white matter development during early childhood
Source: Brain Struct Funct. 2014 Apr 8;220(4):1921–33. doi: 10.1007/s00429-014-0763-3 (PMC4481335; doi:10.1007/s00429-014-0763-3)
Supplement: Supplementary file 7 — Supplementary material 7 (DOCX 76 kb) [file 429_2014_763_MOESM7_ESM.docx]

**Supplementary Table 3:** Mullen scales of early learning information of participants divided by age group. Age group assignment and scores reflect values from the first assessment. A total of 69 subjects were included in the analysis of VF_M_ development and Mullen assessment scores due to a ceiling effect of this assessment battery.

|  | **3 months** | **6 months** | **9 months** | **12 months** | **15 months** | **18 months** | **21 months** |
| --- | --- | --- | --- | --- | --- | --- | --- |
| Participants | 21 | 17 | 8 | 10 | 6 | 2 | 5 |
| Male / Female | 14 / 7 | 9 / 8 | 6 / 2 | 2 / 8 | 4 / 2 | 1 / 1 | 1 / 4 |
| Mullen Fine Motor Raw Score | 5.90 ± 0.94 | 7.79 ± 1.69 | 13.56 ± 1.74 | 16.33 ± 1.07 | 17.67 ± 1.63 | 19.67 ± 1.53 | 22.57 ± 2.51 |
| Mullen Visual Reception Raw Score | 4.48 ± 2.29 | 8.63 ± 2.24 | 12.00 ± 2.18 | 14.67 ± 1.50 | 19.33 ± 3.50 | 20.33 ± 3.51 | 24.57 ± 4.69 |
| Mullen Expressive Language Raw Score | 4.76 ± 1.34 | 7.11 ± 1.29 | 10.33 ± 3.08 | 12.67 ± 1.78 | 15.83 ± 2.71 | 18.67 ± 4.04 | 23.43 ± 4.04 |
| Mullen Receptive Language Raw Score | 4.81 ± 0.87 | 5.46 ± 0.84 | 7.89 ± 2.15 | 11.83 ± 1.85 | 15.00 ± 6.69 | 18.00 ± 4.00 | 20.86 ± 3.76 |
| Mullen Gross Motor Raw Score | 6.22 ± 1.06 | 9.53 ± 1.81 | 12.38 ± 0.74 | 17.45 ± 2.73 | 20.17 ± 1.47 | 22.00 ± 4.24 | 27.00 ± 2.16 |
