# Supplementary material for: Characterizing longitudinal white matter development during early childhood
Source: Brain Struct Funct. 2014 Apr 8;220(4):1921–33. doi: 10.1007/s00429-014-0763-3 (PMC4481335; doi:10.1007/s00429-014-0763-3)
Supplement: Supplementary file 8 — Supplementary material 8 (DOCX 106 kb) [file 429_2014_763_MOESM8_ESM.docx]

**Supplementary Table 4:** Complete results from examining male/female differences. Values in bold denote significant differences between males and females (p<0.05, corrected using Holm-Bonferroni). Regions denoted with NaN correspond to locations where the residuals from the simpler, sex-combined model were smaller than the residuals from the more complex, sex-separated model and thus F-statistics between the male/female trajectories were not applicable. Parameters labeled with * denote regions in which female values were larger than male values, while ** denotes regions where male parameters were larger than female parameters

| **Region/tract** | **Male/Female Trajectory Comparison** | **Male/Female *modified* Gompertz Parameter Comparison** |
| --- | --- | --- |
| Cerebellum WM | 0.1151 | *α* β* γ** δ*** |
| Frontal WM | 0.3225 | *α* β** γ** δ*** |
| Occipital WM | 0.8319 | *α* β* δ*** |
| Parietal WM | 0.6559 | *α* β** γ** δ*** |
| Temporal WM | 0.7326 | *α* β** γ** δ*** |
| Caudate | 0.8865 | *α* β* γ* δ*** |
| Insula | NaN | *α* β** γ** δ*** |
| Putamen | 0.0834 | *α* β** γ** δ*** |
| Thalamus | 0.2515 | *α* β* γ** δ*** |
| Body of Corpus Callosum | 0.6498 | *α* β** γ** δ*** |
| Genu of Corpus Callosum | 0.8187 | *α* β** γ** δ*** |
| Splenium of Corpus Callosum | NaN | *α** β* γ* δ*** |
| Left Cingulum | 0.6279 | *α* β** γ** δ*** |
| Right Cingulum | 0.7083 | *α* β* γ** δ*** |
| Left Anterior Corona Radiata | NaN | *α* β** γ** δ*** |
| Right Anterior Corona Radiata | NaN | *α* β** γ** δ*** |
| Left Posterior Corona Radiata | 0.9716 | *α* β* γ* δ*** |
| Right Posterior Corona Radiata | 0.9973 | *α* β** γ** δ*** |
| Left Anterior Limb of Internal Capsule | 0.8039 | *α* β** γ** δ*** |
| Right Anterior Limb of Internal Capsule | 0.3058 | *α* β** γ** δ*** |
| Left Posterior Limb of Internal Capsule | 0.3493 | *α* β* γ** δ*** |
| Right Posterior Limb of Internal Capsule | 0.5385 | *α* β* γ** δ*** |
| Left Posterior Thalamic Radiation | 0.4841 | *α* β* γ** δ** |
| Right Posterior Thalamic Radiation | 0.1925 | *α* β* γ* δ*** |
| Left Superior Corona Radiata | 0.995 | *α* β** γ** δ*** |
| Right Superior Corona Radiata | NaN | *α* β* γ** δ*** |
| Left Superior Longitudinal Fasiculus | 0.9168 | *α* β* γ** δ*** |
| Right Superior Longitudinal Fasiculus | NaN | *α* β* γ** δ*** |
