# Supplementary material for: Characterizing longitudinal white matter development during early childhood
Source: Brain Struct Funct. 2014 Apr 8;220(4):1921–33. doi: 10.1007/s00429-014-0763-3 (PMC4481335; doi:10.1007/s00429-014-0763-3)
Supplement: Supplementary file 9 — Supplementary material 9 (DOCX 133 kb) [file 429_2014_763_MOESM9_ESM.docx]

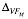

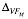
**Supplementary Table 5:** Complete results from longitudinal comparison of the change in VF_M_ and change in raw Mullen assessment scores. Values on the left hand side correspond to the p values from the correlations between and Δ*_M_*, while values on the right hand side correspond to the p values from the examination of the interactions of and Δ*_M_* with mean age.

| **Region/tract** | **Visual Reception** | | **Expressive Language** | | **Receptive Language** | | **Gross Motor** | | **Fine Motor** | |
| --- | --- | --- | --- | --- | --- | --- | --- | --- | --- | --- |
| Cerebellum WM | **0.0001** | 1 | 1 | **0.0111** | **0.0002** | 1 | **0.0061** | 1 | 0.9987 | 0.0972 |
| Frontal WM | 0.2973 | 0.9828 | 0.9951 | 0.4358 | 0.186 | 0.9991 | 0.0606 | 0.9998 | 0.9577 | 0.242 |
| Occipital WM | 0.0592 | 0.9998 | 1 | 0.153 | **0.0176** | 1 | **0.0123** | 1 | 0.9852 | 0.153 |
| Parietal WM | 0.1355 | 0.9986 | 0.9982 | 0.376 | 0.104 | 0.9999 | **0.0312** | 1 | 0.9806 | 0.1645 |
| Temporal WM | 0.2809 | 0.9898 | 0.99 | 0.5133 | 0.1993 | 0.9991 | 0.1083 | 0.9991 | 0.9615 | 0.2162 |
| Caudate | 0.1883 | 0.994 | 0.9998 | 0.3089 | 0.1056 | 0.9997 | **0.0212** | 1 | 0.9482 | 0.2724 |
| Insula | 0.1276 | 0.999 | 0.9998 | 0.2582 | 0.0847 | 0.9999 | **0.0223** | 1 | 0.9703 | 0.206 |
| Putamen | 0.1709 | 0.9964 | 0.9999 | 0.2755 | 0.0789 | 0.9999 | **0.0235** | 1 | 0.9577 | 0.2422 |
| Thalamus | **0.0003** | 1 | 1 | **0.0436** | **0.0023** | 1 | **0.0007** | 1 | 1 | **0.04** |
| Body of Corpus Callosum | 0.1077 | 0.9996 | 0.9985 | 0.3943 | 0.124 | 0.9998 | **0.012** | 1 | 0.9849 | 0.1299 |
| Genu of Corpus Callosum | 0.3835 | 0.9783 | 0.9646 | 0.6462 | 0.2575 | 0.9992 | **0.0485** | 1 | 0.9759 | 0.185 |
| Splenium of Corpus Callosum | 0.0624 | 0.9999 | 0.9956 | 0.4016 | 0.0727 | 0.9999 | **0.0102** | 1 | 0.9987 | 0.0648 |
| Left Cingulum | 0.4193 | 0.933 | 0.9811 | 0.6651 | 0.2829 | 0.9895 | 0.1421 | 0.9986 | 0.9164 | 0.3531 |
| Right Cingulum | 0.4867 | 0.9341 | 0.9701 | 0.6526 | 0.2979 | 0.9946 | 0.1765 | 0.9956 | 0.924 | 0.3321 |
| Left Anterior Corona Radiata | 0.4195 | 0.9637 | 0.9701 | 0.6695 | 0.2702 | 0.9967 | 0.0707 | 1 | 0.9785 | 0.1944 |
| Right Anterior Corona Radiata | 0.3786 | 0.9688 | 0.9872 | 0.5058 | 0.2717 | 0.9964 | 0.0643 | 1 | 0.9449 | 0.2831 |
| Left Posterior Corona Radiata | 0.15 | 0.9961 | 0.9999 | 0.2504 | 0.1036 | 0.9998 | **0.0138** | 1 | 0.9524 | 0.2566 |
| Right Posterior Corona Radiata | 0.1309 | 0.9986 | 0.9986 | 0.3642 | 0.0741 | 0.9999 | **0.0325** | 1 | 0.9596 | 0.2376 |
| Left Anterior Limb of Internal Capsule | 0.0919 | 0.9996 | 0.9999 | 0.2645 | 0.067 | 0.9999 | **0.0143** | 1 | 0.9733 | 0.1887 |
| Right Anterior Limb of Internal Capsule | 0.1072 | 0.9996 | 0.9999 | 0.2361 | 0.0567 | 0.9999 | **0.0103** | 1 | 0.9666 | 0.1987 |
| Left Posterior Limb of Internal Capsule | **0.0001** | 1 | 1 | **0.0429** | **0.0012** | 1 | **0.0007** | 1 | 1 | **0.0173** |
| Right Posterior Limb of Internal Capsule | **0.0001** | 1 | 1 | **0.02** | **0.0009** | 1 | **0.0008** | 1 | 0.9997 | 0.0583 |
| Left Posterior Thalamic Radiation | 0.0858 | 0.9987 | 0.9999 | 0.3018 | 0.0541 | 0.9999 | **0.0109** | 1 | 0.9834 | 0.2041 |
| Right Posterior Limb Internal Capsule | 0.1552 | 0.994 | 0.9999 | 0.31 | **0.0331** | 1 | **0.0169** | 1 | 0.9419 | 0.293 |
| Left Superior Corona Radiata | **0.0353** | 0.9999 | 1 | 0.1471 | **0.0368** | 0.9999 | **0.0015** | 1 | 0.9884 | 0.1562 |
| Right Superior Corona Radiata | **0.0378** | 0.9999 | 1 | 0.1411 | **0.0272** | 1 | **0.0029** | 1 | 0.9692 | 0.194 |
| Left Superior Longitudinal Fasiculus | **0.03** | 0.9999 | 1 | 0.2106 | **0.0333** | 1 | **0.0036** | 1 | 0.9939 | 0.0997 |
| Right Superior Longitudinal Fasiculus | **0.0465** | 0.9999 | 0.9999 | 0.2612 | **0.0427** | 0.9999 | **0.0057** | 1 | 0.9904 | 0.1404 |
